# Interleukin-15 potentiates human natural killer cells to resist tumor-induced suppression through mTOR-regulated metabolic control

**DOI:** 10.1186/2051-1426-3-S2-P232

**Published:** 2015-11-04

**Authors:** Andreas Lundqvist, Yumeng Mao, Xiaonan Zhang, Erik Wennerberg, Vincent Van Hoef, Ola Larsson, Stig Linder, Rolf Kiessling

**Affiliations:** 1 Karolinska Institutet, Stockholm, Sweden

## 

In cancer patients, anti-tumor functions of NK cells are severely impaired by a variety of immunosuppressive mechanisms. Interleukin (IL)-2 and -15 are two essential cytokines regulating the development and function of human natural killer (NK) cells. Here, we compared the role of IL-2 and IL-15 to render resistance of human NK cells to tumor-induced suppression. We found that early-passage melanoma tumor cells strongly inhibited functions of IL-2 activated NK cells through production of prostaglandin E2 (PGE2). Under the same condition, IL-15 activated NK cells could significantly retain the ability to proliferate in vitro durability, in comparison to IL-2-expanded cells. Altogether, our study uncovers distinct properties between IL-2 and IL-15 on primary human NK cells under tumor-induced suppression. It provides evidence that implementation of IL-15 may greatly improve the clinical efficacy of adoptive NK cell therapy for the treatment of human cancers.

